# Towards elimination of maternal deaths: maternal deaths surveillance and response

**DOI:** 10.1186/1742-4755-10-1

**Published:** 2013-01-02

**Authors:** Sennen Hounton, Luc De Bernis, Julia Hussein, Wendy J Graham, Isabella Danel, Peter Byass, Elizabeth M Mason

**Affiliations:** 1 UNFPA Technical Division, New York, USA; 2 Aberdeen University, Aberdeen, UK; 3 US Centers for Disease Control and Prevention, Atlanta, USA; 4 Umea University, Umea, Sweden; 5 WHO, Geneva, Switzerland

## Abstract

Current methods for estimating maternal mortality lack precision, and are not suitable for monitoring progress in the short run. In addition, national maternal mortality ratios (MMRs) alone do not provide useful information on where the greatest burden of mortality is located, who is concerned, what are the causes, and more importantly what sub-national variations occur. This paper discusses a maternal death surveillance and response (MDSR) system. MDSR systems are not yet established in most countries and have potential added value for policy making and accountability and can build on existing efforts to conduct maternal death reviews, verbal autopsies and confidential enquiries. Accountability at national and sub-national levels cannot rely on global, regional and national retrospective estimates periodically generated from academia or United Nations organizations but on routine counting, investigation, sub national data analysis, long term investments in vital registration and national health information systems. Establishing effective maternal death surveillance and response will help achieve MDG 5, improve quality of maternity care and eliminate maternal mortality (MMR ≤ 30 per 100,000 by 2030).

## Introduction

Of all Millennium Development Goals (MDGs), improving maternal health (MDG 5) is the least likely to be achieved. Despite knowledge on determinants and causes, and effective clinical and public health strategies [1], the goal of reducing, the maternal mortality ratio (MMR) between 1990 and 2015 by three quarters is unlikely to be met. More than ever before, maternal and newborn health has received heightened attention from the United Nations, governments, non-governmental organizations, and civil society [2]. The Commission on Information and Accountability of the Global Strategy for Women’s and Children’s Health recommended the implementation of an accountability framework that in countries is based on national oversight, accurate and comprehensive monitoring of results, regular multi-stakeholder review of data and responses, all key features of traditional surveillance and response systems. Significant reduction of maternal mortality in countries will require counting every case and collection of information to permit an effective response that prevents future deaths [3]. Thus a maternal death surveillance and response (MDSR) Technical Working Group (TWG) has been established and chaired by the World Health Organization. The overall objectives of the MDSR are: 1) To provide information that effectively guides actions to eliminate preventable maternal mortality at health facilities and in the community; and 2) To count every maternal death, permitting an assessment of the true magnitude of maternal mortality and the impact of actions taken to reduce it [4]. The upcoming technical guidance of MDSR by the World Health Organization will provide comprehensive concepts of MDSR. This paper describes why MDSR is needed and what would be entailed, builds on lessons from communicable disease surveillance, and considers the pre-requisites for widespread implementation.

### Eliminating maternal mortality

The vision ‘no woman should lose her life when giving birth’ reflects the human rights perspective on maternal mortality and would require that 90% of maternal deaths (when diagnosed and treated in a timely manner) [5], be avoided, making maternal mortality a potential target for an elimination strategy. The term elimination in this paper refers to a significantly lowered level to the point at which maternal mortality ceases to be a major public health burden in countries (for example a goal of a MMR ≤ 30 per 100,000 by 2030 [5] although other target dates such as 2035 or much closer 2025 are being considered).

Although pregnancy is not a disease and maternal death is non-communicable, the good and bad outcomes (births, deaths, complications for mothers and newborns and subsequent disabilities) are all relevant to public health. All issues carrying the burdens of mortality and morbidity warrant public health surveillance systems, and although most have been established for communicable diseases, similar principles could be applied to maternal events [6-8].

### Paradigm shift: why MDSR and what is its added value?

#### Rationale for MDSR

Tracking progress on maternal mortality ratios or rates is notoriously difficult given the lack of reliable vital registration in developing countries and problems in the ascertainment of pregnancy status especially in its early stages. Current national MMR estimates are generated by United Nations agencies and academia despite the absence of civil vital registration and the difficulties in capturing maternal deaths [9-11]. These estimates use aggregated national figures, which lack precision; are not timely, referring to the past; and are often not readily available in formats like simple maps or trend diagrams. Current methods in many developing countries use large-scale periodic surveys (national censuses, Demographic and Health Surveys, Multiple Indicator Cluster Surveys, etc.), which are expensive and data are retrospective and not released in a timely manner. These estimates carry wide confidence intervals and often provide no clues for action. Such estimates are often disputed by countries, and not acted upon. Efforts to generate national estimates have usually ignored the equity dimension of maternal mortality within countries, mainly because of large sample size errors that are associated with survey methodologies for relatively rare events [12]. MDSR would assist in computing national, country-owned maternal mortality data, as well as provide more reliable MMR at sub national levels, thereby showing where the greatest burden of mortality is located, who is concerned and what the causes are. MDSR can inform the actions needed to prevent maternal deaths both in the community and in health facilities. MDSR can improve the quality of care provided to pregnant women by identifying gaps in health services that contributed to a maternal death.

Data are needed to enable short term progress tracking, intervention evaluation, timely actions and increased accountability of civil society, policy makers, managers and donors at national and sub-national levels. These data must be locally relevant, ‘fit for purpose’, inform an immediate response system and be presented in simple table and graphic formats with stories that are powerful and which catalyze action – hence creating accountability. This, together with improvements in health information systems, requires a paradigm shift in counting and responding to maternal deaths instead of solely providing numerical estimates.

#### Added value of MDSR

Public health surveillance involves continuous interpretation of data, essential for the planning, implementation and evaluation of public health practice [13,14]. Surveillance in principle can be either passive or active. Passive surveillance relies on routine reporting, is simple and is minimally burdensome to health care providers, but notification is usually not timely or complete enough to be useful. Active surveillance involves targeted searching for cases, and provides more timely and less variable data, which is clearly needed for maternal deaths.

The immediate added value of active surveillance of maternal deaths would include timely notification of events, assessment and confirmation of cases, increased awareness and advocacy, and most importantly accountability for health services, policy makers, managers and civil society for monitoring progress. The term ‘surveillance’ is not new and has been used with reference to maternal health to address maternal death reviews, audits, confidential enquiries, or at demographic surveillance sites [15-17]. However, converting surveillance systems and responses originally developed for communicable and non-communicable diseases for the purpose of eliminating maternal mortality has only recently been adopted as a framework with guidelines for implementation being developed. Not only is MDSR required for accountability, but a clear pre and post-MDG agenda towards eliminating maternal mortality is also needed such as a proposed goal of MMR ≤ 30 per 100,000 by 2030 [5].

### What would MDSR entail?

#### Pre-requisites for MDSR

First and foremost MDSR requires a mandatory notification of maternal deaths. Steps towards such mandatory notification have taken place in several regions. *The Integrated Disease Surveillance and Response (IDSR)* guidelines have been updated to include maternal deaths. Several sub Saharan African, Asian and Latin American countries (such as South Africa and Tamil Nadu state in India) have adopted mandatory notification of maternal deaths in the first 48 hours, although full implementation in the context of MDSR is yet to be achieved even in these relatively well developed systems.

Second, for epidemic diseases control, most countries have a national management committee for epidemic and outbreak control: The national management committees are typically headed by a high-level Ministry of Health official with convening power, and includes stakeholders from civil society, community leaders, parliamentarians, security, news and media, finance and education, administration, the donor community, international and national NGOs and UN partners. As described in the recommendations of the Commission on Information and Accountability [2], this type of national oversight mechanism for maternal, newborn and child health will recommend remedial actions as required. The terms of reference of the national management committee include the development and validation of surveillance and response strategies, coordination of all partners’ contributions and actions, monitoring and evaluating responses. This multi-sector, data driven and action oriented model of national oversight has been missing in most maternal mortality committees.

#### Establishing a MDSR system

**Figure 1:**
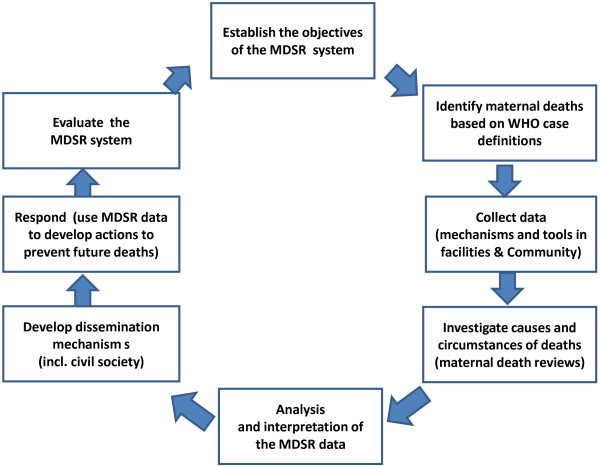
**Steps in Planning Maternal Deaths Surveillance and Response (MDSR) System (Adapted from Teutsch SM [**[14]**] and Gregg M [**[15]**].**

Figure 1 describes classic steps in surveillance and response system (Figure 1) [14].

**First**, define the goals of the system, which is the elimination of maternal mortality. More specifically the system will confirm individual cases, identify causes and underlying factors, monitor levels and trends in maternal deaths and develop standard operating procedures (SOPs) and performance indicators for the system (Table 1).

**Table 1:** Sample performance indicators of a MDSR system*

**Indicators**	**Targets**
**Overall system indicators**	
Maternal death is a notifiable event	Yes
National maternal death review committee exists	Yes
- that meets regularly	At least quarterly
National maternal mortality report published annually	Yes
% of districts with maternal death review committees	100%
% of districts with someone responsible for MDSR	100%
**Identification and notification**	
Health facility:	
All maternal deaths are notified	Yes
% within 24 hours	>90%
Community:	
% of communities with ‘zero reporting’ monthly	100%
% of community maternal deaths notified within 48 hours	>80%
District	
% of expected maternal deaths that are notified	>90%
**Review**	
Health facility	
% of hospitals with a review committee	100%
% of health facility maternal deaths reviewed	100%
% of reviews that include recommendations	100%
Community	
% of verbal autopsies conducted for suspected maternal deaths	>90%
% of notified maternal deaths that are reviewed by district	>90%
District	
District maternal mortality review committee exists	Yes
- and meets regularly to review facility and community deaths	At least quarterly
% of reviews that included community participation and feedback	100%
**Data Quality Indicators**	
Cross-check of data from facility and community on same maternal death	5% of deaths cross-checked
Sample of WRA deaths checked to ensure they are correctly identified as not maternal	1% of WRA rechecked
**Response**	
Facility	
% of committee recommendations that are implemented	>80%
- quality of care recommendations	>80%
- other recommendations	>80%
District	
% of committee recommendations that are implemented	>80%
**Reports**	
National committee produces annual report	Yes
District committee produces annual report	Yes
- and discusses with key stakeholders including communities	Yes
**Impact**	
Quality of care (requires specific indicators, such as case fatality rates)	
District maternal mortality ratio	Reduced by 10% annually
Hospital maternal mortality ratio/lethality rates	Reduced by 10% annually

*To be adapted to each country context.

MDSR will define the guidelines, tools, mechanisms and indicators for its implementation. MDSR will build on existing surveillance and other information systems at country level. It is critical not to create a parallel system, but one which integrates with existing mechanisms of reporting at country level.

**Second,** provide case definitions (Table 2): This is a cornerstone of any surveillance system and although maternal mortality is not a communicable disease the rationale for case definition remains the same. A case definition needs to be simple and sensitive enough to avoid missing cases and should specify criteria for confirmation of cases.

**Table 2:** Maternal death case definitions

**Cases**	**Definitions**
**Death of a woman of reproductive age (WRA):**	Death of woman in reproductive years, usually 15–49 years (although some countries may decide to use other reference period years given the importance of teenage pregnancy and early marriage). All death of WRA should be investigated to determine whether the woman was pregnant or within 42 days of the end of a pregnancy.
**Suspected case**	The death of any woman while pregnant or within 42 days of the termination of pregnancy including deaths where there is a suggestion of a pregnancy even though it may not have been confirmed. In places where the concept of ‘42 days’ may not be well understood the time period can be extended to 2–3 months to ensure that all maternal deaths are captured
**Probable case**	All deaths of women while pregnant or within 42 days of the termination of pregnancy exception of those that are easily determined to be caused by incidental or accidental causes (e.g. motor vehicle accidents)
**Confirmed case**	Probable case with ascertainment of cause of death (either using physician ascertainment of medical records or probabilistic modelling of verbal autopsy) and is defined by a death of a woman while pregnant or within 42 days of pregnancy ending, irrespective of the duration and site of the pregnancy, from any cause related to or aggravated by the pregnancy or its management, but not from accidental or incidental causes*
**Late maternal death**	Death of a woman from direct or indirect obstetric causes more than 42 days but less than one year after termination of pregnancy

*Confirmation of a maternal death is sometimes challenging, particularly for indirect deaths. Final confirmation is generally done by a maternal mortality review committee.

**Third**, develop data collection mechanisms and instruments.

**Figure 2:**
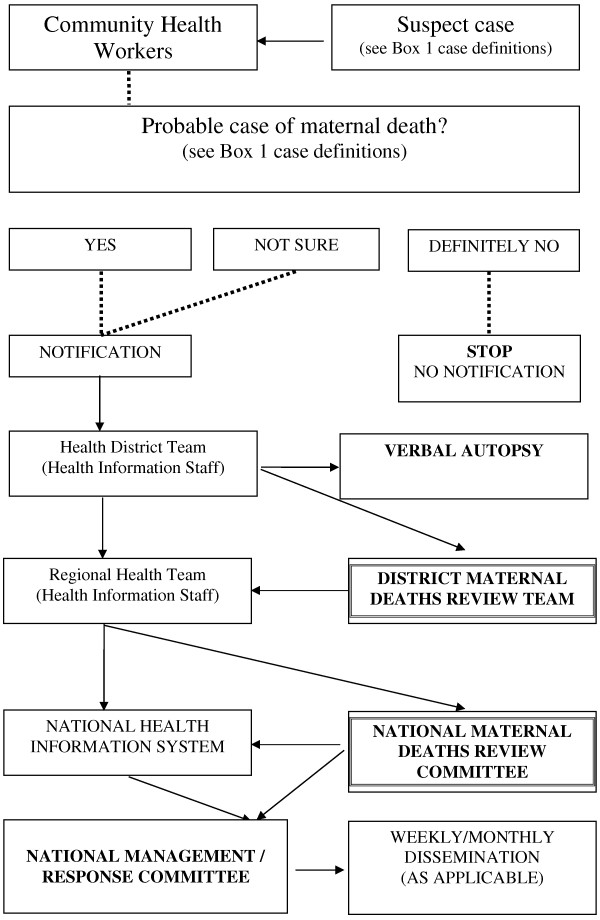
Sample flow diagram or decision tree for national MDSR system (to be adapted based on each country context).

Figure 2 illustrates a sample data collection mechanism (to be adapted based on each country context). Data collection instruments at community level need to be very simple with few data requirements, for example, name of deceased, residence (village, districts, sub national geographic area relevant to the country), time (date of death), place (death in health facility or not), and pregnancy status. Confirmation of the maternal death could be challenging and additional tools will include verbal autopsy, medical files and maternal death review tools. M Gregg [15] suggested the need to ‘remember surveillance is a fluid process – as populations or health problems change…we need to overcome the real tendency to wait or postpone starting a surveillance system until everything is scientifically perfect’. In communicable disease surveillance, data are usually generated from health facilities from the lowest level of the health system to the central level. The MDSR should use the existing facility level notification and reporting systems as well as community health workers from each health facility catchment areas for timely notification and reporting of cases at the community level. There may be a need to improve identification of relevant deaths by introducing a systematic means of recording pregnancy status in women, including teenagers, in any hospital service, rather than confining data collection to maternity units [18]. The use of community health workers and the required timeliness of the maternal death notification will likely involve the use of mobile technologies.

**Fourth**, investigate through maternal death reviews, verbal autopsies and confidential enquiries.

In a communicable disease surveillance, investigations aim to improve speed (to reduce the toll on mortality and morbidity), and certainty (by establishing determinants and confirming the cause) of causal mechanisms including the agent. In maternal and newborn health, speed is not paramount as there is no contagion, but the confirmation of the maternal death, the determination of causes, determinants and how to prevent future cases remain essential and has to be done in a reasonable time frame. The investigation function is fulfilled by verbal autopsies at community level, maternal death reviews at facility level, and including confidential enquiries into maternal deaths within national mechanisms [19]. The overall goal is to ascertain causes of adverse outcomes of pregnancies and to contribute to improvements in quality of care as summarized in the World Health Organization seminal publication ‘Beyond the numbers’ [19]. The investigation is done by analysing cases of death and identifying clinical, community and health systems factors which have to be addressed to avoid subsequent deaths most importantly, at local (community and facility) level and also at regional and national levels [19,20]. The UK confidential enquiry into maternal deaths (CEMD) is one of the oldest systems which has been in place for over 50 years, and has been mooted as one of the reasons behind the UK’s success in reducing maternal mortality [16]. A number of other middle income countries, including Malaysia, Sri Lanka, South Africa, Jamaica and Egypt have successfully used confidential enquiries for several years and demonstrated coincident reductions in maternal mortality [21-23]. The US CDC confidential enquiry system has looked to answer specific questions like racial disparities [17]. In most of these countries, relatively reliable death registration systems are available, which have enabled identification of maternal deaths. In other developing countries, investigation into maternal mortality has also been used, for example in The Gambia [24], in Malawi [25], and sometimes referred to surveillance, such as in India [26]. The lack of a vital registration system will hamper efforts to identify maternal deaths. In countries with poor vital registration systems, maternal death reviews are used primarily at health facilities, but this may result in biases due to mortality outside health facilities, incomplete data retrieval, especially if registers are not well kept, case notes missing or misplaced and independent sources of verification of death not available. Setting up MDSR will benefit the identification of maternal deaths and therefore provide an enabling environment for establishing solid denominators through birth and death registration systems and thus for maternal death reviews and confidential enquiries into all maternal deaths. The committees will determine the factors that contributed to the maternal death and make recommendations to prevent similar deaths in the future.

**Fifth**, analyse and interpret data.

Data analysis and interpretation include a simple line listing, descriptive statistics (people, place and time of maternal deaths) as well as case reviews. Confirmation of cases is essential and will be done for deaths in facilities (using patients’ records) as well as in communities (using verbal autopsy data). The required timeliness of the MDSR is likely to involve probabilistic models of verbal autopsy (http://www.interva.net) [27-29], instead of traditional reliance on physicians to assign cause of death on the basis of VA data (difficult in most remote settings in high maternal mortality countries given scarcity of physicians and transaction costs of finding a third physician evaluation when the first two disagree on causes of death).

**Sixth**, develop dissemination mechanisms.

**Figure 3:**
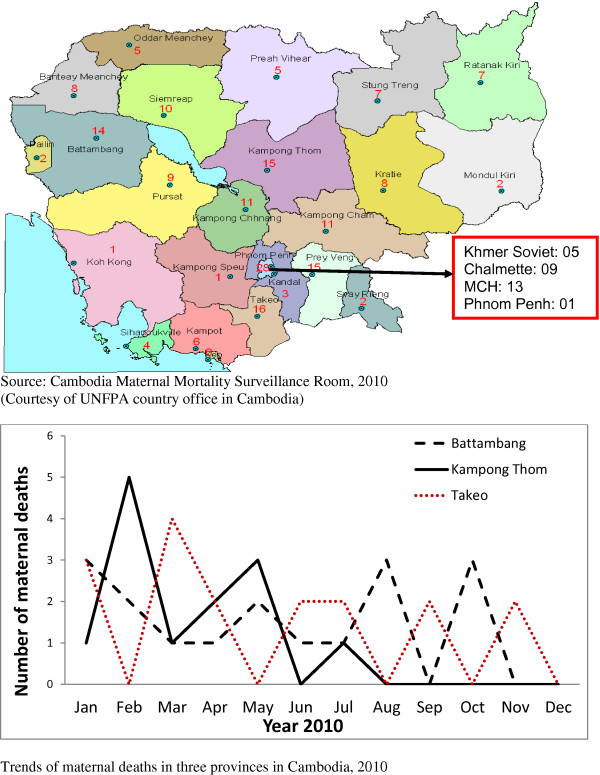
Sample mapping and trends of maternal deaths in Cambodia, 2010.

The availability of data is likely to change the dynamics of advocacy, resources mobilization, accountability, and effectiveness for preventing maternal mortality at national and sub-national levels. Having weekly/monthly/quarterly maternal death trends reported on a regular basis in a prominent national newspaper is a powerful instrument to move the agenda of maternal mortality reduction forward by mobilizing policy makers, but also to improve accountability of governments, communities and professionals (Figure 3). The current existing mechanisms of briefing of high level decision makers should be used to update and engage on numbers of cases, place of occurrence, and causes.

An important dimension whilst developing MDSR is related to communication. It is paramount to plan for communication before the system is established, the communication of results, the management of crisis (unintended consequences such as rebuttal, denial or denigration for political reasons) and communication for advocacy and resource mobilization.

**Seventh**, respond by using the MDSR data to implement preventive actions.

This is the most important step, and given the multiplicity of priorities media and governments are often more responsive to catastrophic and tangible news. Although communities, professionals and governments may agree that levels of maternal mortality are unacceptable, they may not have data that spur timely and routine action, or engage the community. Having regular (monthly, quarterly) figures on maternal deaths at facility or district levels and at national level, reviewed by the district level and national management committee for maternal health can prompt countries to set-up an alert system (Figure 3). This regular information should prompt responsiveness, accountability by comparison across years and areas, and between countries in a region of the world. This real-time data can prompt questions about the need for changes, why a community or health facility cannot do better, what the underlying causes are, or whether effective measures have been put in place and evaluated. Among other key issues, MDSR can identify critical gaps in the quality of care provided to pregnant and post-partum women that must be addressed to prevent maternal death. This is the necessary investment to make maternal mortality elimination a national agenda.

**Eighth**, evaluate the MDSR system.

Given the resources required to set-up and run MDSR, countries should start with basic features, building on existing health information systems. The system will need to be improved subsequently to increase efficiency, and evaluation will identify areas for improvement. In practice, following the classic framework of an evaluation of disease surveillance system is possible. The table below presents sample indicators and performance criteria for the MDSR system and could be adapted to each context. It could include keys steps such as a review of engagement of stakeholders to ensure buy-in and support, the description and review of the operation, attributes (simplicity, flexibility, acceptability, sensitivity, specificity, predictive positive value, representativeness, timeliness), costs, the design of evaluation to be used, and description of data collection, data analysis, report writing, and follow-up of recommendations. The results of the evaluation will be examined by the national oversight committee for maternal and newborn health and appropriate actions will be identified and implemented to improve the surveillance system.

## Conclusion

Presentation, format and accuracy of data are critical for mobilising communities and stakeholders and ensuring accountability for reducing maternal deaths. One of the shortcomings of current maternal mortality estimates is the lack of precision, resulting in presentation of point estimates with large confidence intervals. These estimates are often misinterpreted in comparisons of point estimates of maternal mortality ratios with no reference to confidence intervals. Maternal mortality surveillance and response (MDSR) offers a way forward and will improve accurate identification, counting, and reporting of deaths in settings where vital registration systems have low coverage. Most importantly MDSR will improve current estimations of maternal mortality ratios, will provide data for action to improve quality of care and reduce maternal deaths. MDSR will enhance accountability mechanisms by providing information on whether policies and actions meant to reduce maternal mortality are effective. Having data at sub-national level for action and accountability will also provide better opportunities for equity-based interventions. This paper presents the different components and steps for implementing MDSR. Countries are at various stages of health systems development and will implement MDSR differently based on levels of mortality and health service factors. Regardless of the variations in countries’ contexts, accountability requires a bold investment towards maternal mortality elimination with a possible goal of an MMR ≤ 30 per 100,000 by 2030 [5]. Implementing and improving completeness of MDSR and establishing solid denominators through birth and death registration systems will be achieved over time, but a journey that does not get started can never be completed.

## Competing interests

The authors declare that they have no competing interests.

## Authors’ contributions

SH conceived and proposed the original draft. All authors designed, and participated in the writing and reviewing of the final manuscript. All authors read and approved the final manuscript.
